# Precise Loran-C Signal Acquisition Based on Envelope Delay Correlation Method

**DOI:** 10.3390/s20082329

**Published:** 2020-04-19

**Authors:** Wenhe Yan, Kunjuan Zhao, Shifeng Li, Xinghui Wang, Yu Hua

**Affiliations:** 1 National Time Service Center, Chinese Academy of Sciences, Xi’an 710600, China; 2 University of Chinese Academy of Sciences, Beijing 100049, China; 3 Key Laboratory of Precise Positioning and Timing Technology, Chinese Academy of Sciences, Xi’an 710600, China

**Keywords:** Loran-C, signal acquisition, envelope delay correlation, cross-rate interference

## Abstract

The Loran-C system is an internationally standardized positioning, navigation, and timing service system. It is the most important backup and supplement for the global navigation satellite system (GNSS). However, the existing Loran-C signal acquisition methods are easily affected by noise and cross-rate interference (CRI). Therefore, this article proposes an envelope delay correlation acquisition method that, when combined with linear digital averaging (LDA) technology, can effectively suppress noise and CRI. The selection of key parameters and the performance of the acquisition method are analyzed through a simulation. When the signal-to-noise ratio (SNR) is −16 dB, the acquisition probability is more than 90% and the acquisition error is less than 1 μs. When the signal-to-interference ratio (SIR) of the CRI is −5 dB, the CRI can also be suppressed and the acquisition error is less than 5 μs. These results show that our acquisition method is accurate. The performance of the method is also verified by actual signals emitted by a Loran-C system. These test results show that our method can reliably detect Loran-C pulse group signals over distances up to 1500 km, even at low SNR. This will enable the modern Loran-C system to be a more reliable backup for the GNSS system.

## 1. Introduction

The Loran-C system is an internationally standardized radio positioning, navigation, and timing (PNT) service system. It has the advantages of high transmission power, long propagation distance, and good phase stability [1,2,3]. It can provide a time service better than 100 ns and a position service of 20 m after differential correction. The signal can be transmitted up to a distance of 1500 km [4,5]. When a signal from the global navigation satellite system (GNSS) is rejected or interfered with, Loran-C can be used as an important backup system of GNSS navigation and timing [2,6]. Loran-C can also be effective for “timing warfare” and “navigation warfare” in modern war [7]. Therefore, many countries are developing and upgrading their Loran-C systems to improve their accuracy, availability, and integrity. In June 2017, the U.S. Congress passed the Frank LoBiondo Coast Guard Authorization Act of 2018. The bill formally restarted the Loran-C system to improve the security and reliability of position and timing systems. The goal is to achieve accurate and reliable Loran-C navigation and timing services in 50 states by building or rebuilding Loran-C time service stations [8,9,10]. China also plans to build three Loran-C stations in Western China in 2018 to provide national signal coverage with the existing Loran-C system. In 2013, South Korea completed preparations for the construction of its Loran system with the aim of upgrading its Loran-C stations to an autonomous Loran-C system, hoping to achieve full coverage of the country in the future [11,12]. In summary, the Loran-C system will develop vigorously in the future. The Loran-C signal processing technology also needs further development; improving the accuracy and coverage of system applications will serve to meet the application needs of modern PNT services.

The Loran-C signal acquisition involves finding the first pulse position of the master and secondary stations without manual intervention. This is a key step in signal processing performed by navigation and timing receivers [13]. With the increase in the number of Loran-C stations, the cross-rate interference between the stations will become increasingly pronounced. At the same time, the electromagnetic environment is becoming increasingly complex. Noise and interference, whether intentional or unintentional, also affect the detection of Loran-C signals. Owing to the limitations of digital signal processing technology (DSP), the traditional receiving system generally adopts hard-limiting technology [14,15]. This results in quantization of the received Loran-C signal into a one bit digital signal, and acquisition is realized by determining the strict group repetition interval (GRI) and pulse phase codes characteristics of the Loran-C signal. The accuracy of phase code detection and recognition is the key to signal acquisition. Accurate detection of phase codes needs a sufficient signal-to-noise ratio (SNR). In the case of strong noise and interference, the probability of signal acquisition is low, which reduces the effective range of Loran-C receivers [16,17]. With the development of DSP, several acquisition methods have been recently developed. In [13], an acquisition method based on the matched filter method is proposed. This method uses the local standard Loran pulse signal and the received pulse signal to achieve acquisition by matching correlation. However, this method does not consider the influence of noise and cross-rate interference (CRI) on the Loran-C pulse signal. When the Loran-C signal is influenced by CRI and noise, the received signal is not a standard Loran-C signal. The influence of matched filtering of this method becomes worse, which leads to the decrease of acquisition accuracy and probability. In [16], the delay correlation acquisition method based on the Loran-C pulse signal is used. In this method, the received Loran-C pulse signal is delayed by one GRI and then correlated with the next group of pulse signals to realize the acquisition of the pulse group. This method can suppress the effect of CRI interference. In the case of a high SNR, this method can effectively detect the pulse group signal. In the case of a low SNR, this method results in a decrease in the correlation, and the signal acquisition is difficult. Moreover, the signal of the master and secondary stations cannot be simultaneously detected.

Therefore, this article proposes an envelope delay correlation (EDC) acquisition method, combined with linear digital averaging (LDA) and adaptive threshold techniques, which can effectively reduce the effect of noise and CRI on the signal acquisition process. Furthermore, the correlation peaks can also be repaired by this method, so that the acquisition results have higher accuracy. The method in this article can accurately and simultaneously detect the signals of the master and secondary stations, thus improving the sensitivity and effective range of Loran-C receivers.

## 2. Materials and Methods

### 2.1. Loran-C Signal Format

**Figure 1:**
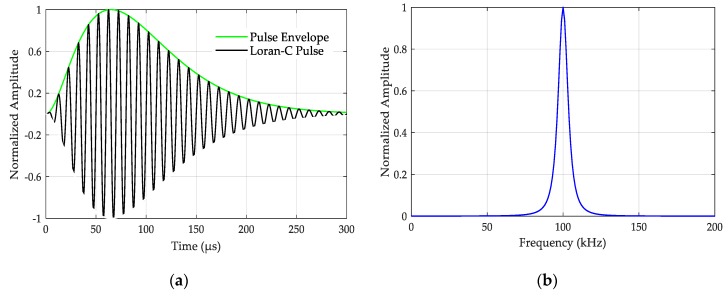
The characteristics of a single Loran-C signal for a positive phase-coded pulse: (**a**) Loran-C pulse waveform; (**b**) Loran-C pulse spectrum.

The Loran-C signal is formally defined by the United States Coast Guard (USCG) as a sequence of pulses of radio frequency (RF) energy at a central frequency of 100 kHz [18]. A single Loran-C pulse is defined by

(1) $$S\left(t\right)=\{\begin{array}{cc} 0 & for\;t<\tau \\ E\left(t\right)\sin(\omega_{0}t+P_{c}) & for\;\tau \leq t\leq 65+\tau \\ undefined & for\;t>65+\tau \end{array},$$

where $E\left(t\right)=A{\left(t-\tau \right)}^{2}\exp\left(\frac{-2\left(t-\tau \right)}{65}\right)$ is the Loran-C pulse envelope, A is a normalization constant related to the peak amplitude, t is the time in μs, τ is the envelope-to-cycle difference (ECD) in μs, $\omega_{0}=2\pi f_{0}$, $f_{0}=100$ kHz, and $P_{c}$ is the phase code parameter (in radians), which is 0 for positive phase-coded pulses and π for negative phase-coded pulses. The vast majority (90%) of the energy of Loran-C signals is concentrated in the frequency band of 90–110 kHz. This frequency band is reserved for Loran-C and it should be free from any intentional interference. The characteristics of a single Loran-C signal are shown in Figure 1.

**Figure 2:**
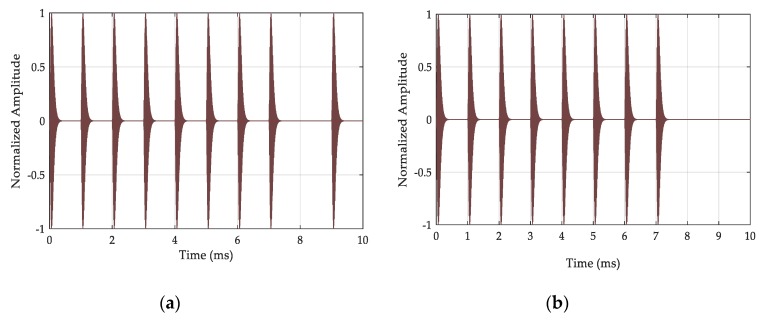
The characteristics of a Loran-C pulse group signal: (**a**) master station; (**b**) secondary station.

The Loran-C navigation chain is composed of one master station and two to five secondary stations. The pulse groups signal of the master and secondary stations are shown in Figure 2. The pulse group signals of the master station transmits nine Loran-C pulse signals. The interval between the first eight pulses is 1 ms, and the interval between the eighth and ninth pulses is 2 ms. The secondary station only transmits the first eight pulses, and the Loran-C timing system only transmits the signal of the master station [1,19]. The phase code cycles of Loran-C pulse group signals are shown in Table 1. The two code cycles are alternately modulated with the signal: “+” indicates a phase code of 0 radians and “−” indicates a phase code of π radians.

**Figure 3:**
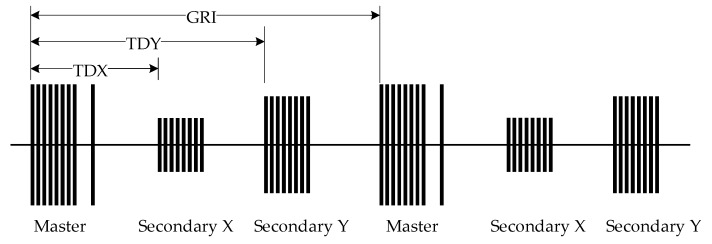
The signal transmission format of Loran-C navigation chains. Abbreviations: group repetition interval (GRI); time delay for first secondary station (TDX); time delay for second secondary station (TDY).

The interval between the Loran-C pulse groups is called the group repetition interval (GRI) [20]. The GRI cycle range is defined as 40,000–99,990 μs with a resolution of 10 μs. The Loran-C system continuously emits the pulse group signals according to its own GRI. The signal transmission format of Loran-C navigation chains is shown in Figure 3. The master station transmits the pulse group signal first. After the specified time delay (denoted by TDX), the first secondary station transmits the pulse signal. After the time delay TDY, the second secondary station transmits the pulse signal. Finally, after a time delay equal to the GRI, the master station retransmits the pulse group signal, and the cycle repeats.

### 2.2. Loran-C Interference Sources

The inherent interference in Loran-C signals is mainly from noise and cross-rate interference [21,22]. They have different effects on the amplitude and acquisition accuracy of Loran-C pulse group signals. These effects are described in this subsection.

Noise is an integral part of any radio system, and it can originate from sources external to the receiving system or from within the receiving system itself. External sources include lightning discharges, precipitation, electronics and power transmission. The main internal noise source is thermal noise generated in the receiver antenna and front end [21,23]. The SNR of the received Loran-C signal is one of the key factors in determining the usefulness of Loran-C signals for navigation and timing. Noise exists at all times and can have a significant effect on the SNR of Loran-C signals. As the signal propagation distance increases, the signal level decreases and the SNR also decreases, which makes it difficult to detect the signal. At the same time, it also causes the signal waveform to change, which reduces the signal detection accuracy. Therefore, it is necessary to ensure that the pulse group signal can be detected at low SNR.

**Figure 4:**
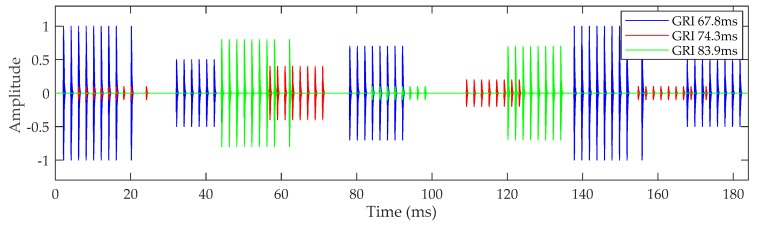
Schematic diagram of cross-rate interference (CRI).

Although each Loran-C station has a different GRI, all Loran-C transmitters transmit their signals with the same frequency and format. Therefore, the signal is often interfered with by signals from other stations in the navigation chain. This is called cross-rate interference (CRI) [21,24]. A schematic diagram of CRI is shown in Figure 4. CRI signals will occur periodically, causing distortion of the received signals. CRI is inherent to the Loran-C system and cannot be prevented. Nevertheless, if the CRI signal is not suppressed, the acquisition result will be incorrect (especially when the CRI is intense), and the receiver will not receive the required signal [25,26]. With the increasing number of Loran-C stations being built, the amount of CRI is also increasing. Therefore, it is essential for the CRI to be suppressed during the process of pulse group detection.

### 2.3. Loran-C Acquisition Method

#### 2.3.1. Process of the Acquisition Method

**Figure 5:**
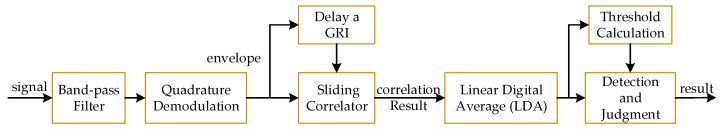
Flow diagram of the signal acquisition method used in this article.

The first step in the signal detection process is the acquisition of Loran-C signals, which enables the starting position of the master and secondary pulse group signals to be found. In this article, Loran-C pulse group signals are detected using the envelope delay correlation (EDC) method. This acquisition method is shown in Figure 5 and outlined below.

**Step 1:** The received signal passes through a band-pass filter (BPF) to remove interference and noise outside the band. Its bandwidth is 30 kHz. 

**Step 2:** The signal envelope is obtained through quadrature demodulation. The signal envelope is delayed by a GRI, and the correlation peak is obtained using the EDC method, which can reduce the influence of CRI.

**Step 3:** The correlation peak is accumulated and averaged according to the GRI period by using linear digital averaging (LDA) technology, which can restore the Loran-C correlation peak and reduce the influence of interference on the delay correlation peak amplitude.

**Step 4:** The detection threshold is calculated according to the statistical characteristics of the noise in the correlation results. Based on the characteristics of the Loran-C pulse group signal, the correlation peak amplitude is compared with the detection threshold. With this, the acquisition of the master and secondary pulse group signal is achieved.

#### 2.3.2. Envelope Delay Correlation (EDC)

We assume that the digital Loran-C signal, after passing through the band-pass filter, is

(2) $$S_{i}\left(n\right)=\pm A_{r}\left(nT_{s}\right)\sin\left(2\pi f_{0}nT_{s}\right)+N_{i}\left(nT_{s}\right),$$

where $T_{s}=\frac{1}{f_{s}}$ is the sample period, $f_{s}$ is the sample frequency, $A_{r}\left(nT_{s}\right)$ is the signal envelope, $\sin\left(2\pi f_{0}nT_{s}\right)$ is the signal carrier, $f_{0}$ is the signal frequency of 100 kHz, ± is the pulse phase code, and $N_{i}\left(nT_{s}\right)$ is the noise. The received signal is then mixed with the orthogonal carrier according to [27,28]:

(3) $$S_{I}\left(n\right)=S_{i}\left(nT_{s}\right)\cos\left(2\pi f_{0}nT_{s}+\Delta \emptyset_{n}\right)\;=\mp \frac{1}{2}A_{r}\left(nT_{s}\right)\left[\sin\left(\Delta \emptyset_{n}\right)-\sin\left(2\pi f_{0}nT_{s}+\Delta \emptyset_{n}\right)\right]+N_{i}\left(nT_{s}\right)\cos\left(2\pi f_{0}nT_{s}+\Delta \emptyset_{n}\right)$$

(4) $$S_{Q}\left(n\right)=S_{i}\left(nT_{s}\right)\sin\left(2\pi f_{0}nT_{s}+\Delta \emptyset_{n}\right)\;=\pm \frac{1}{2}A_{r}\left(nT_{s}\right)\left[\cos\left(\Delta \emptyset_{n}\right)-\cos\left(2\pi f_{0}nT_{s}+\Delta \emptyset_{n}\right)\right]+N_{i}\left(nT_{s}\right)\sin\left(2\pi f_{0}nT_{s}+\Delta \emptyset_{n}\right),$$

where $S_{I}\left(n\right)$ and $S_{Q}\left(n\right)$ are the result of mixing. $\Delta \emptyset_{n}$ is the phase difference between the received signal and the orthogonal carrier signal. The above results were filtered with a low-pass filter to obtain two orthogonal signals [29,30]:

(5) $$I\left(n\right)=\mp \frac{1}{2}A_{r}\left(nT_{s}\right)\sin\left(\Delta \emptyset_{n}\right)+N_{I}\left(nT_{s}\right),$$

(6) $$Q\left(n\right)=\pm \frac{1}{2}A_{r}\left(nT_{s}\right)\cos\left(\Delta \emptyset_{n}\right)+N_{Q}\left(nT_{s}\right),$$

where I(n) and Q(n) are the results of low-pass filtering. The high frequency components in Equation (3) and Equation (4) can be filtered using a low-pass filter. The signal envelope can be obtained with

(7) $$E_{r}\left(n\right)=\sqrt{I^{2}\left(nT_{s}\right)+Q^{2}\left(nT_{s}\right)}+N^{\prime }\left(nT_{s}\right),$$

where $N^{\prime }\left(nT_{s}\right)$ is the noise that exists after processing. The above equation gives a positive pulse envelope. This not only eliminates the phase difference θ between the received signal and the orthogonal carrier, but also eliminates the impact of the Loran-C pulse phase code. The Loran-C signal envelope is delayed by one GRI, and sliding correlation is performed with the next set of signal envelopes [31]. The correlation results are

(8) $$R\left(n\right)=\frac{1}{L}\sum_{k=n-L+1}^{n}E_{r}\left(kT_{s}\right)E_{r}\left(kT_{s}-T_{GRI}^{D}\right),$$

where $T_{GRI}^{D}$ is the GRI of the receiving station and L is the sliding correlation time (in μs). Because the correlation peak has the same GRI repetition characteristic of the Loran-C signal, there is a strong correlation between the signal envelopes. The signal envelope of the Loran-C signal is weakly correlated with the noise. In addition, the noise is weakly correlated with itself, and therefore it can be ignored here. R(n) can be alternatively expressed as

(9) $$R\left(n\right)=\frac{1}{4L}\sum_{k=n-L+1}^{n}A_{r}\left(kT_{s}\right)A_{r}\left(kT_{s}-T_{GRI}^{D}\right)+N_{o}\left(nT_{s}\right)=\frac{1}{4L}\sum_{k=n-L+1}^{n}A_{r}^{2}\left(kT_{s}\right)+N_{o}\left(nT_{s}\right),$$

where $N_{o}\left(nT_{s}\right)$ is the noise after the sliding correlation is performed. In Equation (9), the selection of the correlation length L is critical because it can be used to obtain an ideal correlation peak. We obtained correlation peaks in seven cases with *L* values of 32, 64, 96, 128, 160, 192, and 224 μs. The correlation result is shown in the Figure 6.

As can be seen from Figure 6, when L < 64 μs, the correlation peak is very small. As *L* increases, the amplitude of correlation peak also increases. However, when *L* > 128 μs, the correlation peaks are wider and the detection results are multivalued. Therefore, we use a value of *L* = 96 μs, which maximizes the correlation peak amplitude while also ensuring the sharpness of the peak. This is beneficial for signal sensitivity and detection accuracy.

#### 2.3.3. Linear Digital Averaging (LDA) 

The Loran-C signal is easily affected by noise during signal propagation and reception, which causes the envelope correlation peak to be distorted. Therefore, we use the LDA technique to accumulate and average the correlation peaks according to the GRI of the Loran-C signal. LDA not only restores the signal envelope, but also improves the SNR, which stabilizes the changes in the correlation peak. We define the vector representation of the envelope correlation to be

(10) $$H_{i}=\left[r\left(1\right),r\left(2\right),r\left(3\right),\ldots ,r\left(j\right)\right],$$

where $H_{i}$ is the vector form of the correlation result and r(j) is the quantized value of the correlation results. Assuming

(11) $$SNR_{in}=20\log_{10}\left(\frac{A_{i}}{\sigma_{i}}\right),$$

where $A_{i}$ is the amplitude of the signal, and $\sigma_{i}$ is standard deviation of noise. The linear digital average is expressed as

(12) $$Y=\left[b\left(1\right),b\left(2\right),b\left(3\right)\ldots ,b\left(j\right)\right]=\frac{1}{M}\left(H_{1}+H_{2}+H_{3}\ldots .+H_{M}\right),$$

where *M* is the number of accumulations. From [32], the standard deviation of the noise after linear digital averaging is $\sigma_{0}=\frac{\sigma_{i}}{\sqrt{M}}$. Therefore, the output SNR is:

(13) $$SNR_{out}=20\log_{10}\left(\frac{A_{i}}{\sigma_{0}}\right)=10\log_{10}\left(M\right)+SNR_{in}.$$

**Figure 7:**
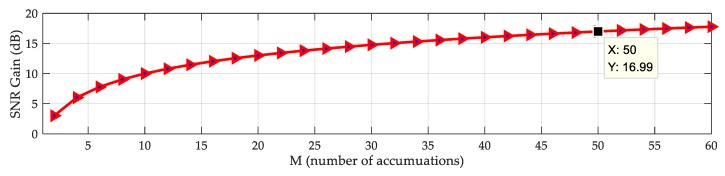
Signal-to-noise ratio (SNR) gain as a function of the number of accumulations.

The above equation indicates that the SNR can be increased by $10\log_{10}\left(M\right)$ through the linear digital averaging method. Figure 7 shows the relationship between the SNR gain and M. After accumulating 50 times, the SNR is increased by 16 dB. Considering the acquisition time and the resources of the receiver, we chose a value of 30 accumulations so that the SNR can be increased by more than 10 dB.

#### 2.3.4. Threshold and Detection 

In order to effectively detect the position of the pulse group signals, the detection threshold needs to be adjusted as the noise amplitude changes. Thus, the threshold needs to be assigned based on the noise amplitude in the LDA results. We calculate the maximum value X(i) of the correlation peak every 1 ms in the correlation accumulation results. This generates $⎣\frac{GRI}{1\;ms}⎦$ values, which are sorted from largest to smallest. The Loran-C navigation chain is composed of one master station and two to five secondary stations. We assume that there are N secondary stations. When signals are received at each station, 9+8N peaks are generated, and the values after the (9+8N)th peak are all noise. Therefore, we use noise values to calculate the threshold. The mean and standard variance of the noise are

(14) $$\bar{X}=\frac{\sum_{k_{1}}^{k_{2}}X\left(i\right)}{k_{2}-k_{1}+1}$$

(15) $$\sigma_{n}=\sqrt{\frac{\sum_{k_{1}}^{k_{2}}{\left(X\left(i\right)-\bar{X}\right)}^{2}}{k_{2}-k_{1}+1}},$$

respectively, where $k_{1}=9+8N+1$ and $k_{2}=⎣\frac{GRI}{1\;ms}⎦$. The detection threshold, then, is defined as

(16) $$V_{t}=\bar{X}+{\alpha \sigma }_{n}^{2},$$

where α is the threshold coefficient, which can vary from 2 to 10 according to the situation [33]. In this article, the threshold is set according to the statistical value of the noise correlation value. The purpose is to allow the threshold value to be independent of changes in the amplitudes of the signals from the master and secondary stations. There are two conditions to detect the pulse group signals successfully: the correlation peak must be greater than the threshold and the characteristics of the master and secondary pulse signals must be present.

## 3. Results 

### 3.1. Validation Method 

**Figure 8:**
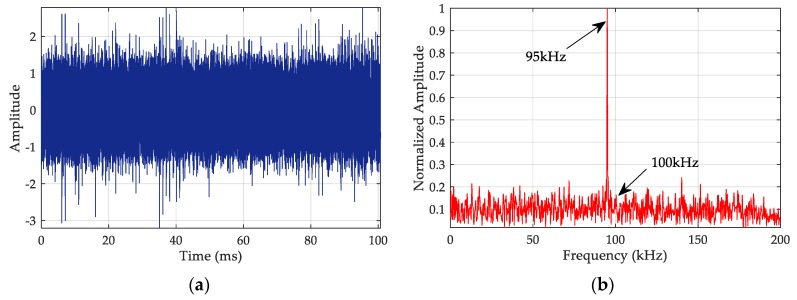
Characteristics of simulated signals: (**a**) signal waveform; (**b**) signal spectrum.

To verify the effectiveness of the proposed method, we performed a simulation, the parameters of which were set as follows. The GRI of the received signals, including the signal of one master and two secondary stations, is 67.8 ms. The CRI, including that of one master station and two secondary stations, is 74.3 ms. The signal-to-interference ratio (SIR) defined as [21]

(17) $$\mathrm{SIR}=20log_{10}\left(\frac{A_{s}}{A_{c}}\right),$$

where $A_{s}$ is the amplitude of the received signal and $A_{c}$ is the amplitude of the CRI. The SIR is set to 0 dB. The continuous wave interference frequency is 95 kHz, and its SIR is 3 dB. The noise is assumed to have the characteristics of white gaussian noise (WGN), and its SNR is 0 dB. The waveform and spectrum are shown in Figure 8.

The waveform of the simulated signal (Figure 8a) shows that the signal has been completely submerged in noise and interference. The spectrum (Figure 8b) clearly indicates that the power of the interference at 95 kHz is very strong and that the signal at 100 kHz is undetectable. Next, we filter and extract the envelope of the signal.

**Figure 9:**
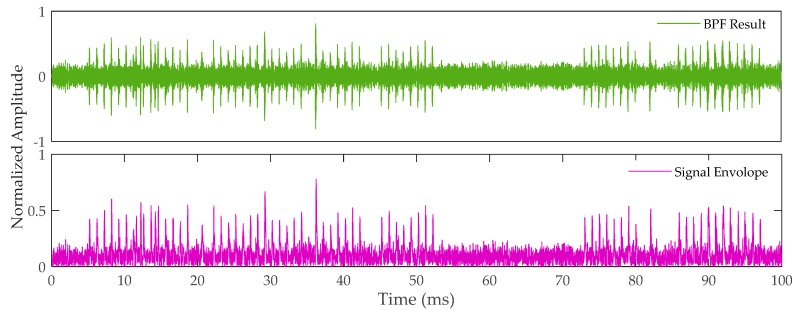
Band-pass filter (BPF) result and signal envelope.

Figure 9 shows the waveform of the simulated signal after band-pass filtering (top panel) and the signal envelope that is obtained through quadrature demodulation (bottom panel). The signal and the envelope can be distinguished, but there is severe CRI. The noise is still strong, which causes the signal amplitude to change rapidly. We now use the acquisition method presented in this article to process the signal.

**Figure 10:**
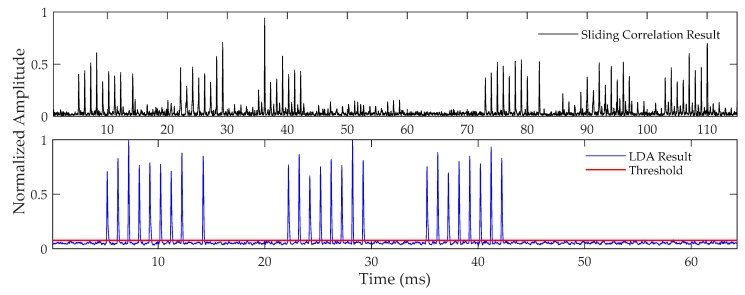
Correlation result and linear digital averaging (LDA) result.

Figure 10 shows the results after the envelope delay correlation (top panel) and linear digital averaging of the signal (bottom panel). The delayed correlation results show that the CRI signal is significantly suppressed. However, there is pulse interference, which significantly modifies the correlation peak amplitude and noise floor. After LDA, the correlation peak is obvious and the noise floor is uniformly distributed. By comparing the correlation result with the threshold, the master and secondary pulse groups can be accurately detected at the same time. This demonstrates the effectiveness of our method.

### 3.2. Anti-noise Performance 

Noise is the most basic source of interference in the received signal, and it is also inevitable. Generally, the receiving system works normally when the SNR is greater than −10 dB [34]. Therefore, in order to verify the anti-noise performance and the acquisition probability and accuracy of our method, pulse group signals with −20 dB < SNR < −5 dB were generated using a simulation.

**Figure 11:**
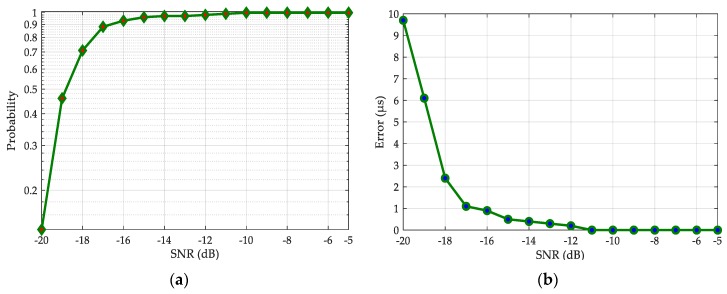
The acquisition performance as a function of SNR: (**a**) acquisition probability; (**b**) acquisition error.

Figure 11 shows the simulation results for the acquisition probability (Figure 11a) and acquisition error (Figure 11b). When the SNR is less than −16 dB, the acquisition performance drops rapidly. However, when the SNR is greater than −16 dB, the acquisition probability of our method reaches more than 90%, and the acquisition error remains within 1 μs. Therefore, our method has excellent anti-noise performance and can meet the minimum requirements of Loran-C receivers.

### 3.3. Cross-rate Interference Effect

CRI is caused by overlapping signals from different Loran-C chains. This will become more severe as the number of stations increases. When the interference signal overlaps with the expected signal, the waveform of the expected signal is affected. The receiving system is required to work normally when the intensity of the CRI signal is lower than the intensity of the expected signal [34]. This section analyzes the influence of CRI on the correlation peak and acquisition accuracy, and evaluates the ability of the acquisition algorithm to suppress CRI. The GRI of the interference signal is set to 40–100 ms, the GRI of the expected signal is 70 ms, and the SIR is assigned a range from −10 dB to 10 dB. The simulation results are shown below.

**Figure 12:**
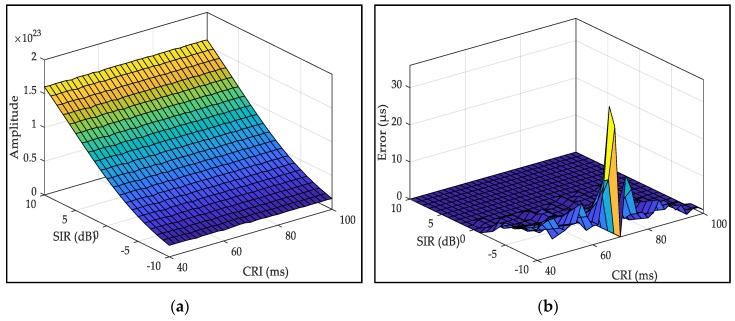
Effect of CRI on correlation peak amplitude and acquisition accuracy: (**a**) amplitude of correlation peak; (**b**) acquisition error.

Figure 12a demonstrates that for the same SIR, the influence of CRI on the expected signal amplitude is the same. As the SIR decreases, the signal amplitude decreases significantly. This indicates that the influence of CRI on the amplitude is related to the SIR. Figure 12b shows that the acquisition error is within 5 μs when the SIR is greater than −5 dB, but when the GRI of the interference signal is close to the GRI of the expected signal, the acquisition error increases significantly due to the increase in the interference rate. Therefore, the simulation results demonstrate that the method in this article can effectively suppress the influence of CRI on the acquisition of pulse groups and that it can meet the requirements of the Loran-C receiving system. 

### 3.4. Experimental Verification

**Figure 13:**
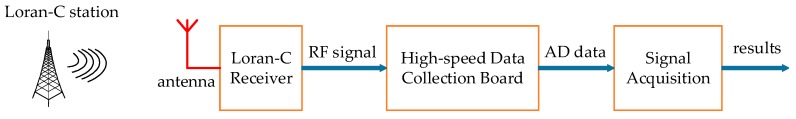
Test scheme of acquisition method.

We test the acquisition method with signals actually transmitted by a real Loran-C system. The test scheme is shown in Figure 13. The Loran-C receiver obtains the Loran-C signals through an antenna, and after processing the signals, the receiver outputs RF signals. We use a high-speed data collection board to collect and save RF signals. The data was processed using the acquisition method presented in this article.

The actual collected signal is the signal emitted by the BPL timing system in China. The coordinates of the BPL timing system are $\left(034^{\circ }57^{\prime }\;N,109^{\circ }33^{\prime }\;E\right)$. Its GRI is 60 ms and its transmission power is 2 MW. Six test sites within 1500 km of the system were selected for data collection. The coordinates, great circle distance, and electric field strength of the test sites are shown in Table 2. Test sites 1–5 are used to collect signals in an open area, and test site 6 is used to collect data indoors.

**Figure 14:**
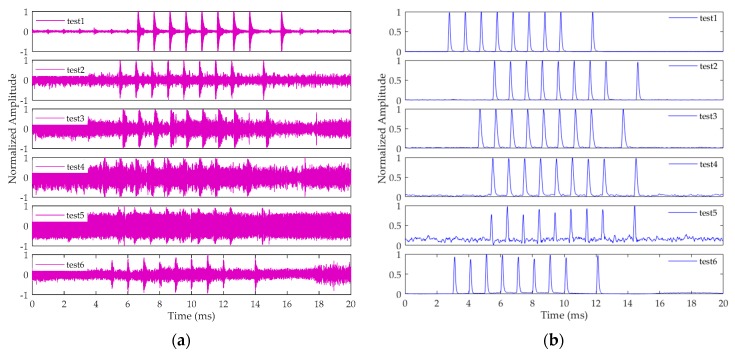
Actual signal and acquisition results of the method in this paper at test sites 1–6: (**a**) signal waveform; (**b**) correlation peak.

Figure 14a shows the signal waveforms at all six test sites. Figure 14b shows the correlation peaks of the signals processed by the method in this article. As can be seen from the figures, the Loran-C signal amplitude gradually decreases as the distance increases. Within 1000 kilometers, the signal waveform is detectable and the Loran-C pulse group can be clearly seen, making the correlation peak of the pulse sharp and obvious. In test site 1, the CRI signal is visible, and the waveform of the correlation peak is not affected. For great circle distances greater than 1000 kilometers, the amplitude of the signal is significantly reduced, and even submerged in noise. However, the pulse group signal can still be easily detected using the acquisition method in this article. The test results show that the method proposed in this article can detect the Loran-C pulse group signals reliably, even at low SNR. The method has engineering application value due to its ability to perform well over distances up to 1500 km.

**Figure 15:**
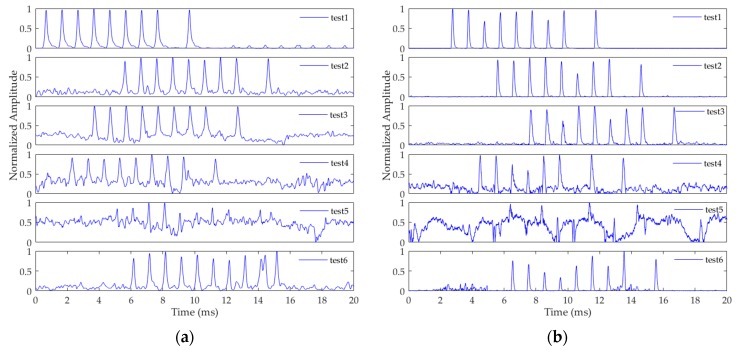
Test results of existing methods with respect to actual data: (**a**) the matched filter method based on Loran-C pulses signal [13]; (**b**) the delay correlation acquisition method based on Loran-C pulses signal [16].

In order to compare the method in this article with the existing method, we use the existing method to process the collected data. Figure 15 shows the results of testing the existing method with respect to actual data. Figure 15a shows the matched filtering method based on Loran-C pulses signal in [13]. As shown in the results, in test 1, the CRI cannot be effectively suppressed by this method. If the amplitude of the received CRI signal is relatively large, the signal acquisition was impacted. Moreover, when the SNR was low, owing to the distortion of the received Loran-C signal, the correlation peak after the matched filtering was not sufficiently large, and there were significant variations in the correlation peak amplitude. Figure 15b shows the delay correlation acquisition method based on the Loran-C pulses signal in [16]. As shown in the figure, this method can suppress the CRI, and when the SNR is high, the method demonstrated an excellent performance. However, with a decrease in the SNR, the performance decreased rapidly, and the pulse group signal was undetectable. Therefore, compared with these two methods, the proposed method demonstrates a remarkable performance under the conditions of a high CRI and low SNR.

## 4. Discussion

The Loran-C system is a remote land-based radio system that can provide high-precision PNT services. However, the traditional Loran-C acquisition method is unable to effectively suppress noise and CRI. Therefore, with the development of modern Loran-C systems, more advanced Loran-C signal processing capacity is required. In this article, we proposed a Loran-C acquisition method based on the EDC method and analyzed the selection of key parameters in the method. In addition, LDA technology was used to restore the correlation peaks and improve the SNR. This article also described the method of calculating the adaptive detection threshold. We demonstrated that our acquisition method can detect the pulse group signals of the master and secondary Loran-C stations at the same time.

In addition, a simulation was used to verify the effectiveness of the acquisition method and analyze its anti-noise and anti-interference performance. The simulation results show that this method can quickly acquire the master and secondary station signals. When the SNR > −16 dB, the acquisition probability reaches more than 90% and the acquisition error is less than 1 μs. When the SIR of the CRI is −5 dB, the method can still detect the pulse group signal effectively because of its ability to suppress interference. Finally, we used our acquisition method to process actual received signals. Those results proved that our method can reliably detect Loran-C pulse group signals over distances up to 1500 km, even at low SNR. Thus, the method has high engineering application value and it is optimized for the design of the new Loran-C navigation and timing receivers. This will improve the performance of the modern Loran-C system, making it a more reliable backup for the GNSS system.

## Figures and Tables

**Figure 6:**
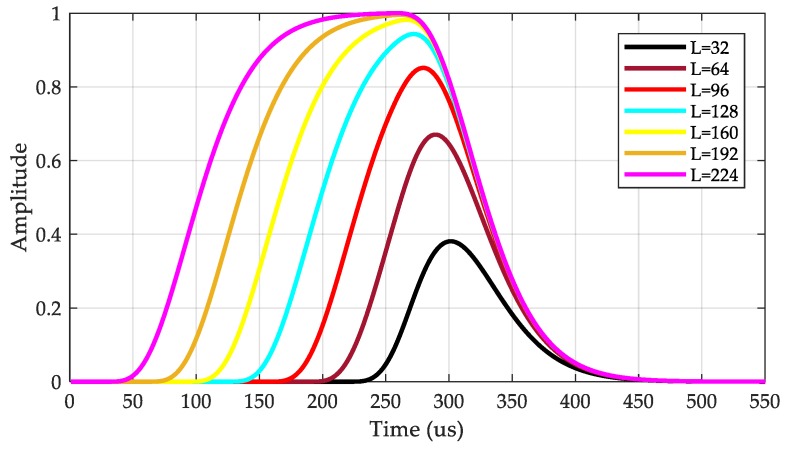
Correlation peaks at different correlation lengths.

**Table 1:** Phase codes of Loran-C signals.

Ground	Master	Secondary
A	+ + − − + − + − +	+ + + + + − − +
B	+ − − + + + + + −	+ − + − + + − −

**Table 2:** Data for the six test sites.

Test	Coordinate	Great Circle Distance (km)	Electric Field Strength (dBμV/m)
1	$\left(35^{\circ }59^{\prime }20.89^{\prime \prime }\;N\;,106^{\circ }11^{\prime }53.43^{\prime \prime }\;E\right)$	325.4	62.9
2	$\left(37^{\circ }55^{\prime }57.85^{\prime \prime }\;N\;,102^{\circ }43^{\prime }49.96^{\prime \prime }\;E\right)$	695.1	59.5
3	$\left(38^{\circ }52^{\prime }44.07^{\prime \prime }\;N\;,100^{\circ }30^{\prime }36.67^{\prime \prime }\;E\right)$	915.5	51.9
4	$\left(40^{\circ }17^{\prime }48.09^{\prime \prime }\;N\;,097^{\circ }05^{\prime }39.18^{\prime \prime }\;E\right)$	1248.0	48.1
5	$\left(41^{\circ }31^{\prime }14.78^{\prime \prime }\;N\;,095^{\circ }39^{\prime }02.21^{\prime \prime }\;E\right)$	1441.3	41.5
6	$\left(034^{\circ }22^{\prime }6.84^{\prime \prime }\;N\;,109^{\circ }13^{\prime }18.32^{\prime \prime }\;E\right)$	71.2	60.4

## References

[1] Qiu D., Boneh D., Lo S., Enge P. (2010). Reliable Location-Based Services from Radio Navigation Systems. Sensors.

[2] Griffioen J.W., Oonincx P.J. (2013). Suitability of Low-Frequency Navigation Systems for Artillery Positioning in a GNSS Denied Environment. J. Navig..

[3] Strategy for the Department of Defense Positioning, Navigation and Timing (PNT) enterprise—Ensuring a US Military PNT Advantage. https://rntfnd.org/wp-content/uploads/DoD-PNT-Strategy.pdf.

[4] Li Y., Hua Y., Yan B.R., Guo W. (2019). Experimental Study on a Modified Method for Propagation Delay of Long Wave Signal. IEEE Antennas Wirel. Propag. Lett..

[5] Wang X.Y., Zhang S.F., Sun X.W. (2017). The Additional Secondary Phase Correction System for AIS Signals. Sensors.

[6] Williams P., Basker S., Ward N. (2008). e-Navigation and the case for eLoran. J. Navig..

[7] Kim H., Lee J., Oh S.H., So H., Hwang D.H. (2019). Multi-Radio Integrated Navigation System M&S Software Design for GNSS Backup under Navigation Warfare. Electronics.

[8] Johnson G., Wiggins M., Swaszek P.F., Hartshorn L., Hartnett R. Possible optimizations for the US Loran system. Proceedings of the 2006 IEEE/ION Position, Location and Navigation Symposium.

[9] Offermans G., Bartlett S., Schue C. (2017). Providing a Resilient Timing and UTC Service Using eLoran in the United States. Navig. J. Inst. Navig..

[10] S.140-Frank LoBiondo Coast Guard Authorization Act of 2018. https://www.congress.gov/bill/115th-congress/senate-bill/140.

[11] Hu A.P., Gong T. (2016). Research Status and Progress on the Enhance Loran-C Navigation Technology. GNSS World Chin..

[12] Joon H.R., JiWon S. (2013). eLoran Signal Strength and Atmospheric Noise Simulation over Korea. J. Position. Navig. Timing.

[13] Gao Y.Y., Hua Y., Li S.F., Yang C.Z. Acquisition method of Loran-C signal based on matched filter. Proceedings of the 2015 IEEE International Conference on Signal Processing, Communications and Computing.

[14] Fisher A.J. (2000). Loran-C cycle identification in hard-limiting receivers. IEEE Trans. Aerosp. Electron. Syst..

[15] Vanderwal P.W., Vanwilligen D. (1978). Hard Limiting and Sequential Detection Applied to Loran-C. IEEE Trans. Aerosp. Electron. Syst..

[16] Li S.F., Wang Y.L., Hua Y., Yuan J.B. (2013). Loran-C Signal Fast Acquisition Method and Its performance Analysis. J. Electron. Inf. Technol..

[17] Zhu Y.B., Cao K.J., Li B. (2012). Study on Auto-Search Algorithm Based on Subsection Correlation for Loran-C. Appl. Mech. Mater..

[18] U.S. Coast Guard and the U.S. Coast Guard Auxiliary Loran-C User Handbook. https://www.loran.org/otherarchives/-1992%20-Loran-C%20User%20Handbook%20-%20USCG.pdf.

[19] Lo S.C., Peterson B.B., Enge P.K. (2007). Loran data modulation: A primer. IEEE Aerosp. Electron. Syst..

[20] Nieuwland A.K. (1995). GRI Ranking Based on Cross-Rate Interference in Loran-C. J. Navig..

[21] Safar J. (2014). Analysis, Modelling and Mitigation of Cross-Rate Interference in Enhanced Loran. Ph.D. Thesis.

[22] Liatos P., Hussein A.M. (2005). Characterization of 100-kHz noise in the lightning current derivative signals measured at the CN tower. IEEE Trans. Electromagn. Compat..

[23] Manish L., Frank G., Curtis C., David D.D. Characterization of Atmospheric Noise in the LORAN-C Band. Proceedings of the International Loran Association (ILA)—32nd Annual Convention and Technical Symposium.

[24] Safar J., Vejrazka F., Williams P. (2012). Accuracy Performance of eLoran Receivers under Cross-Rate Interference Conditions. Annu. Navig..

[25] Bayat M., Madani M.H. (2017). Analysis of Cross-Rate Interference Cancelation by Use of a Novel Phase Code Interval in Loran Navigation System. Navig. J. Inst. Navig..

[26] Safar J., Paul W., Sally B., Frantisek V. Cross-Rate Interference and Implications for Core eLoran Service Provision. Proceedings of the International Loran Association (ILA)—38nd Annual Convention and Technical Symposium.

[27] Kong S.H. (2017). High Sensitivity and Fast Acquisition Signal Processing Techniques for GNSS Receivers from fundamentals to state-of-the-art GNSS acquisition technologies. IEEE Signal Process. Mag..

[28] Geiger B.C., Vogel C., Soudan M. (2012). Comparison Between Ratio Detection and Threshold Comparison for GNSS Acquisition. IEEE Trans. Aerosp. Electron. Syst..

[29] Hinedi S., Statman J. (1990). Digital accumulators in phase and frequency tracking loops. IEEE Trans. Aerosp. Electron. Syst..

[30] Thacker T., Boroyevich D., Burgos R., Wang F. (2011). Phase-Locked Loop Noise Reduction via Phase Detector Implementation for Signal-Phase Systems. IEEE Trans. Ind. Electron..

[31] Yang M.H., Wu H., Wang Q.Q., Zhao Y.X., Liu Z.Y. (2020). A BeiDou Signal Acquisition Approach Using Variable Length Data Accumulation Based on Signal Delay and Multiplication. Sensors.

[32] Richard G.L. (2004). Understanding Digital Signal Processing.

[33] Guo C.L., Gao L., Yu L.J. (2019). A Pulse Detection Method Based on Judgment Threshold Automatic Recommendation. Fire Control & Command Control.

[34] Minimum Performance Standards for Marine eLORAN Receiving Equipment Radio Technical Commission for Maritime Services. https://rtcm.myshopify.com/collections/maritime-navigation-equipment-standards/products/copy-of-differential-gnss-package-both-of-the-current-standards-10402-3-and-10403-3.

